# Genetic polymorphism of Merozoite Surface Protein-2 (MSP-2) in *Plasmodium falciparum* isolates from Pawe District, North West Ethiopia

**DOI:** 10.1371/journal.pone.0177559

**Published:** 2017-05-19

**Authors:** Hussein Mohammed, Moges Kassa, Ashenafi Assefa, Mekonnen Tadesse, Amha Kebede

**Affiliations:** Bacterial, Parasitic and Zoonotic Diseases Research Directorate, Ethiopian Public Health Institute, Addis Ababa, Ethiopia; Centro de Pesquisas Rene Rachou, BRAZIL

## Abstract

**Background:**

In malaria endemic regions, *Plasmodium falciparum* infection is characterized by extensive genetic diversity. Describing this diversity provides important information about the local malaria situation. This study was conducted to evaluate the extent of genetic diversity of *P*. *falciparum* in Pawe district, North West Ethiopia, using the highly polymorphic merozoite surface protein 2 gene.

**Methods:**

Atotal of 92 isolates from patients with uncomplicated *P*. *falciparum* attending Pawe Health Centre were collected from September to December 2013. Genomic DNA was extracted using the Chelex method and analyzed by length polymorphism following gel electrophoresis of DNA products from nested PCR of *msp2* (block 3), targeting allelic families of FC27 and 3D7/IC.

**Results:**

There were twenty-two different *msp2* alleles, 11 corresponding to the 3D7/ IC and 11 to the FC27 allelic family. The frequency of isolates of the *msp2* 3D7/IC allelic familywas higher (51%) compared to FC27 (49%). The majority of the isolates (76%) contained multiple infections andthe overall mean multiplicity of infection was 2.8 (CI 95% 2.55–3.03). The heterozygosity index was 0.66 for *msp2*. There was no statically significant difference in the multiplicity of infection by age or parasite density.

**Conclusions:**

The results of this study show that *P*.*falciparum* polymorphismsare extensive in Northwest Ethiopia and most of the infections are composed of multiple clones.

## Introduction

Despite declining trends, malaria remains an important global public health problem. According to the World Malaria Report 2015, there were about 214 million cases of malaria and an estimated 438,000 deaths. Of these, mostly (90%) occurred in sub-Saharan Africa [1]. Malaria is a major public health problem despite of relatively low malaria prevalence compared to other malaria endemic African countries. As a result, malaria reported confirmed cases were about 1,867,059 (84.1 percent) and an estimated 662 deaths; out of which, 64% were *Plasmodium falciparum* and 36% were *Plasmodium vivax* [2]. However, approximately 60% of the population lives in areas at risk of malaria [3]. Among malaria parasite species, *Plasmodium falciparum* (*P*. *falciparum*) is the most virulent and associated with the most malaria-related deaths [4]. Screening of the diversity of malaria parasite populations is important in monitoring the deployment of malaria control and elimination strategies. Patients infected with multiple parasite clones commonly reside in high transmission intensity areas, which impacts upon the host immune status [5]. In addition, while the development of an effective malaria vaccine would be an important additional tool towards malaria control and elimination, antigenic diversity is a major challenge in its development [6].

Themerozoitesurfaceprotein (MSP-1) and (MSP-2) are of *P*.*falciparum* are associated with an immune response and have been identified aspotential targets for ablood-stage malaria vaccine [7]. MSP2 is a glycoprotein expressed on the surface of merozoites that has been considered as a candidate malaria vaccine [8]. The *msp2* gene has two major allelic families, FC27 and 3D7/IC based on variation in length and sequence in the central region [9]. The *msp2* block 3 is the most polymorphic and could serve alone in describing the diversity of parasite populations. *P*. *falciparum* parasite populations are often characterized by extensive genetic diversity in areas with high transmission intensity [10]. Therefore, investigating the diversity of *P*. *falciparum* and multiplicity of infection (MOI) may be useful to describe the level of malaria transmission [11,12] and to assess the effect of control interventions in the study area.

The genetic diversity of *P*. *falciparum* has been extensively studied in various parts of the world [13–15], but limited data are available from Ethiopia. The only study conducted by Mohammed *et al*. demonstrated moderate to high genetic polymorphism of *P*. *falciparum* populations in southwest Ethiopia which revealed moderate transmission in the area [16]. Since then, no other study has reported the genetic diversity of *P*. *falciparum* in the country. Since that time, malaria prevention and intervention programs have been scaled up [17]. Hence, it is important to reassess the malaria situation by detecting the existing parasite population dynamics, through assessment of parasite genetic diversity, in order to better inform control measures. This study aims to assess the extent of genetic diversity in *P*. *falciparum* isolates from Pawe northwest, Ethiopia.

## Materials and methods

### Study site

**Fig 1:**
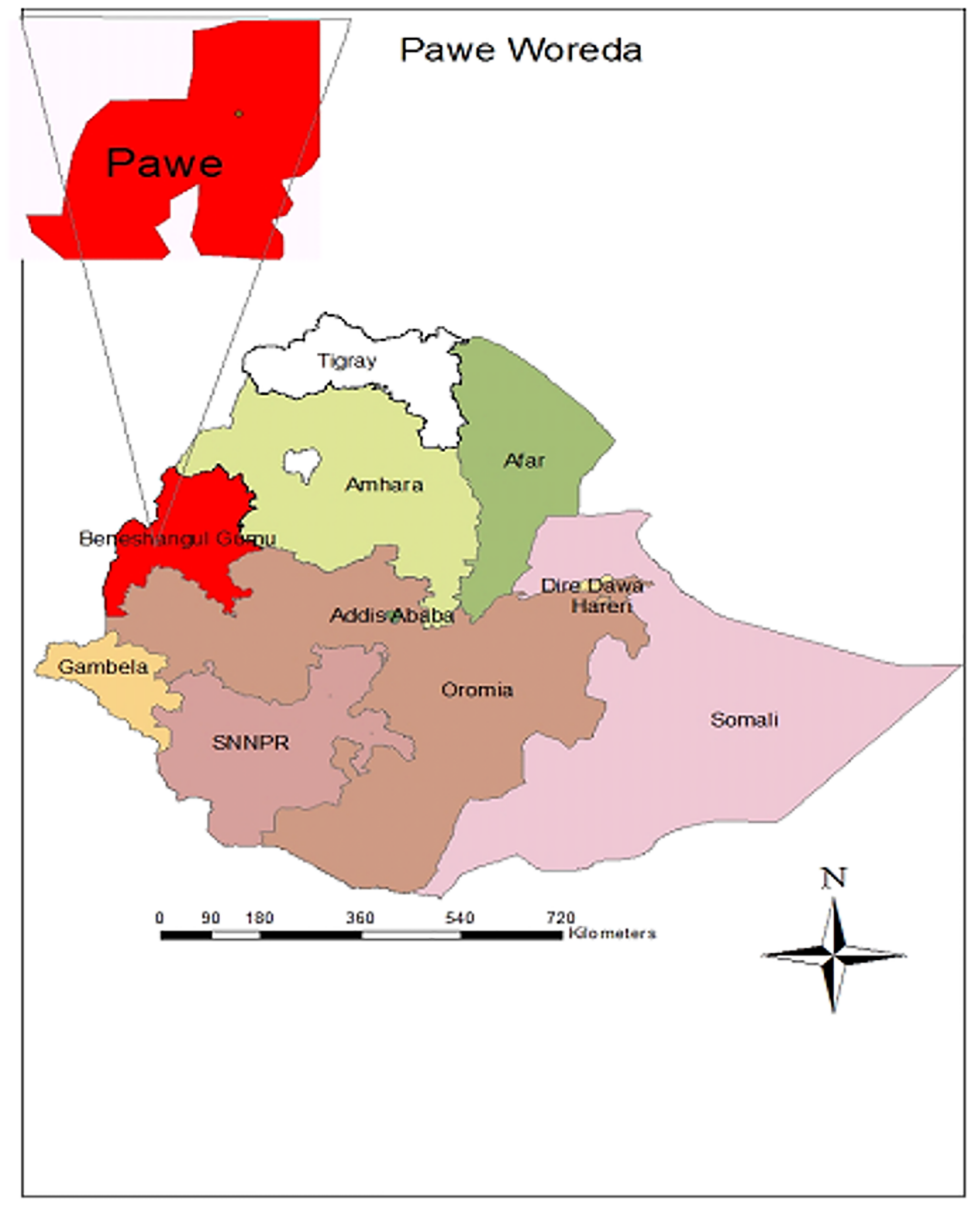
Map of the sample collection area, Pawe district North West Ethiopia.

The study sample was conducted in the rural town of Pawe, Benishangul-Gumuz Regional state in the northwest of Ethiopia (Fig 1). The study area is located 556 km away from Addis Ababa with a catchment population estimated at 45,000 inhabitants. The area is located at an altitude of 1050 meters above sea level with a mean annual temperature ranging from 16.2°C to 32.2°C, and a mean annual rainfall between 980 and 1200 mm occurring in two seasons from March to May and from June to December. Water for mosquito breeding is available from three sources; the Belese river, Ali-spring and Diga dam. Malaria is present throughout the year, with a peak during the rainy season. In this region, *P*. *falciparum* is the dominant malaria species.

### Study population and blood sample collection

A total of 92 *P*. *falciparum* infected blood spot samples were collected during a therapeutic efficacy study ofarthemeter-lumefantrine (Coartem^®^) from September to December, 2013. The samples used in this study were from patients aged three to 60 years, presenting to the local Health Center. Inclusion criteria: all patients consented, were febrile, axillary temperatures ≥37.5°C and positive for asexual *P*. *falciparum* andwereresidents withinthecatchmentarea. The blood was spotted onto 903^®^filter paper (Schleicher & Schuell BioScience), air-dried and individually placed into plastic bags with desiccant, before being transported to the Malaria Research Laboratory (Ethiopian Public Health Institute) at Addis Ababa and kept at -20°C prior to molecular analysis.

### Laboratory procedures

#### Parasite DNA extraction

Genomic DNA was extracted from dried blood spots using the Chelex-100^®^ (Bio-Rad Laboratories CA) method [18], with a final volume of 200 μl for each sample and storage at-20°C until allelic typing.

#### Allelic typing of *P*. *falciparummsp2* gene

The polymorphic region from the *P*. *falciparummsp2* gene block 3 was used as a genetic marker for the genotyping of parasite populations. Genotyping the *msp2* gene was undertaken in a two-step reaction, primary amplification of the entire polymorphic gene segments of block 3, followed by a nested reaction targeting the allelic type-specific regions within this block [19]. All reactions were carried out in a final volume of 20 μl containing 125mM dNTP, 250 nM of each primer, 2mM MgCl_2_, and 0.02 units per ul of Taq polymerase (Roche Applied Science, Germany) and PCR buffer (950mM KCl, 20mMTris-HCl, pH 8.8). In the first round reaction, 4μl of genomic DNA was added as a template. In the nested reaction, 2μl of the first PCR product was added. Each amplification profile consisted of initial denaturation at 94°C for 3 min, followed immediately by 30 cycles at 94°C for 1 min; 50°C for 35 seconds, and 68°C for 2.5 minutes. The final cycle was prolonged extension at 72°C for 3 min. PCR reaction mixtures were incubated in a thermal cycler (MyCycler-BioRad, Hercules, USA). Allelic specific positive control 3D7 and DNA-free negative controls were included in each set of reactions. The nested PCR productsseparated on 2% ethidium bromide-stained agarose gels were visualized under ultraviolet (UV) trans-illumination and photographed. The fragment size was estimated in relation to a 50 bp DNA ladder (Boehringer Mannheim Marker VI). Presence of more than one genotype was considered to be a polyclonal infection, while the presence of a single allele was considered as a monoclonal infection. Alleles in each family were considered the same if fragment sizes were within a 20 bp interval [20]. Heterozygosity index (He), was calculated by using the following formula: He = [n/ (n-1)] [(1-Σpi2)], where n is the number of isolates sampled and pi is the allele frequency at a given locus [21].

### Ethics consideration

The study was ethically approved by the Scientific and Ethical Review Office (SERO) of the Ethiopian Public Health Institute (EPHI). Written informed consent was obtained from the parents or guardian prior to recruitment.

### Data analysis

Data were analyzed using SPSS version 16 (SPSS Inc., Chicago, IL, USA). The *msp2* allelic frequency was calculated as the proportion of the allelic family out of the alleles detected in the isolates. The multiplicity of infection (MOI) was estimated by the average number of PCR fragments per infected individual. Proportion was compared for significance using the Chi-square test. Spearman's rank correlation coefficient was calculated to assess the possible associations between MOI and geometric mean parasite density and age. *P* value of ≤ 0.05 was considered indicative of statistical significance.

## Results

### Demographic and parasitological data

**Fig 2:**
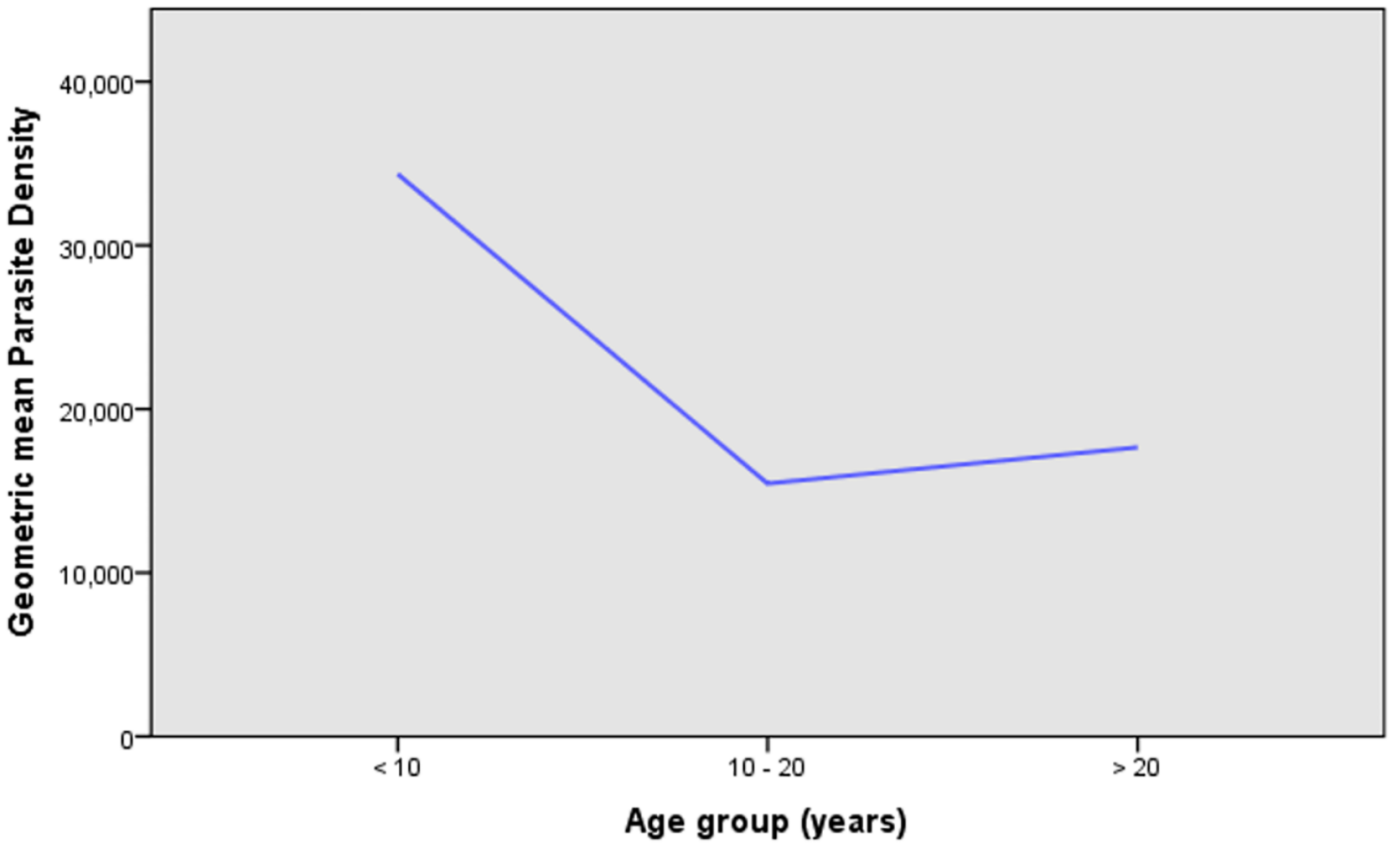
Relationship between geometric mean parasite density and age group (n = 92).

Of the total 92 samples, 54 (58.7%)were from males and38 (41.3%)were from females. The patients’ ages ranged from three years to 60 years (mean age: 15.78 ± 1.062 years). The asexual parasitaemia for *P*. *falciparum* collected samples ranged between 237 parasites to 380,270 parasites/ μl of blood with a geometric mean of 20,491 (95% CI: 15,121–28,354) parasites per μl as shown in Table 1. Children less than 10 years of age had the highest mean parasite density (70,617 parasites / μl ± 15,419)compared to other age groups (Fig 2). The mean parasite density in adults greater than 20 years was 46,229 parasites / μl (± 17,676) (Table 2).

**Table 1:** Demographic and parasitological data of the study individuals at Pawe district, North West Ethiopia, 2016.

Characterization of patients	Values
Mean age (year)	15.78 ± 1.062*
Sex ratio (Male/Female)	1.4 (54/38)
Geometric mean of parasite density (p/μl)	20,491(95% CI: 15,121–28,354)
Parasite density range (p/μl)	237–380,270

**p/μl**: parasite per microliter,

*:Standard deviation.

**Table 2:** Mean of *Plasmodium falciparum* density (parasite/ul) and multiplicity of infection in *msp2* gene stratified by age group (N = 92).

Age (years)	N	Parasite density	MOI
**<10**	**29**	**70,617**	**2.62**
**10–20**	**42**	**40,611**	**3.02**
**>10**	**21**	**46229**	**2.62**

**N**: number of malaria cases, MOI: multiplicity of infection

### Allelic diversity of *Plasmodium falciparummsp2* gene

**Fig 3:**
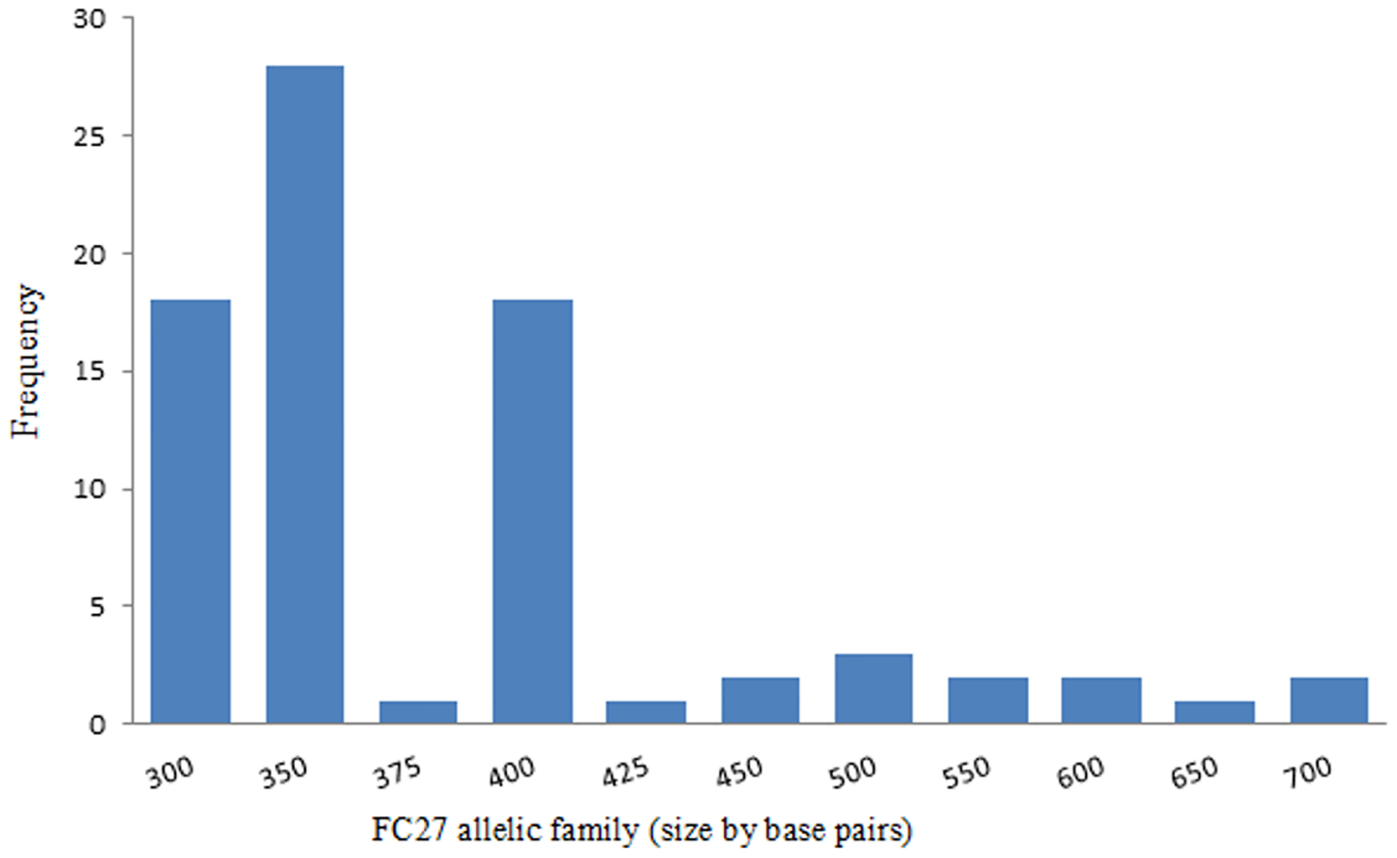
Distribution of FC27 alleles of *P*. *falciparum msp2* gene in isolates in northwest Ethiopia.

**Fig 4:**
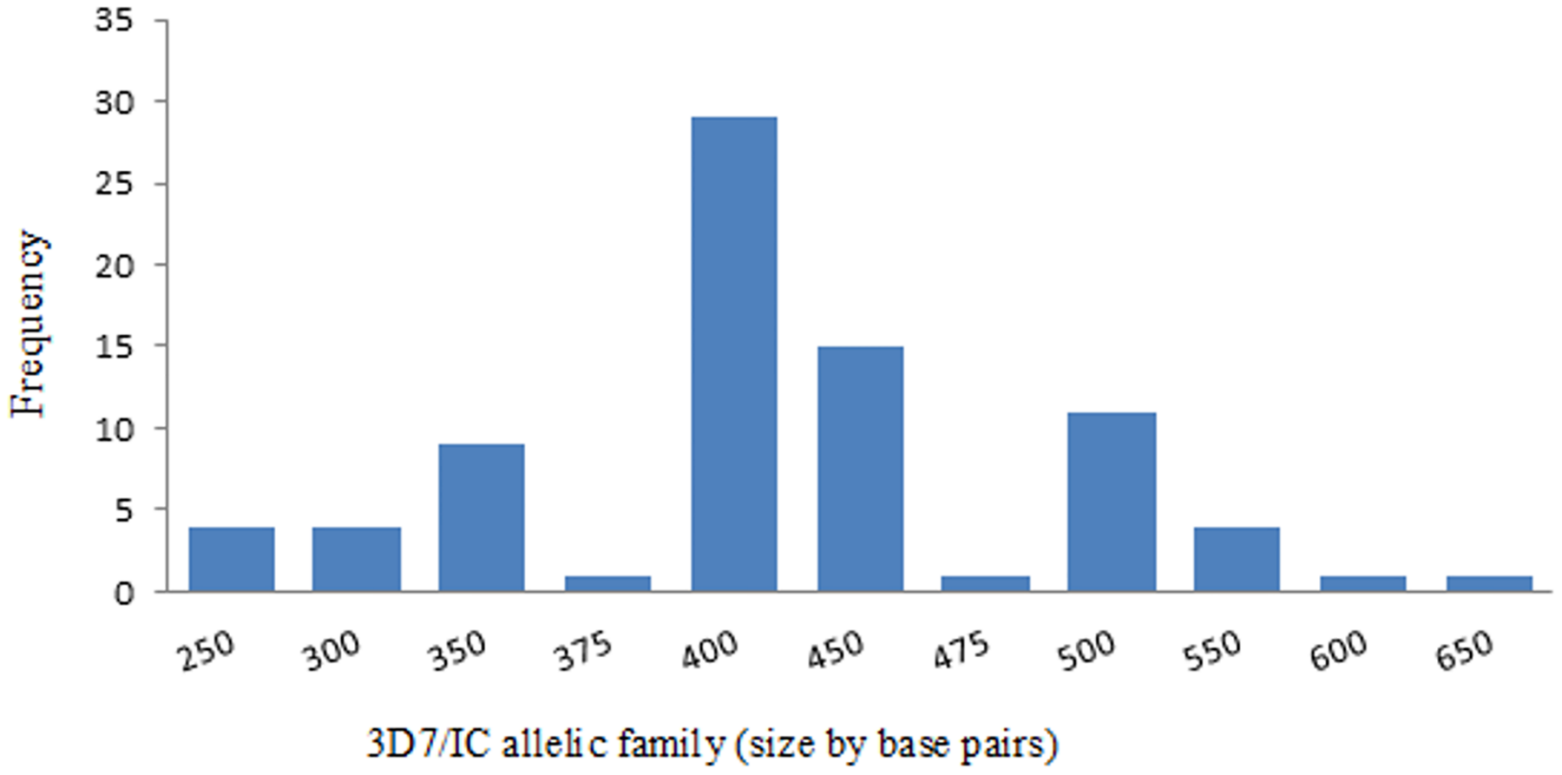
Distribution of 3D7/IC alleles of *P*. *falciparum* msp2 gene isolates in northwest Ethiopia.

**Fig 5:**
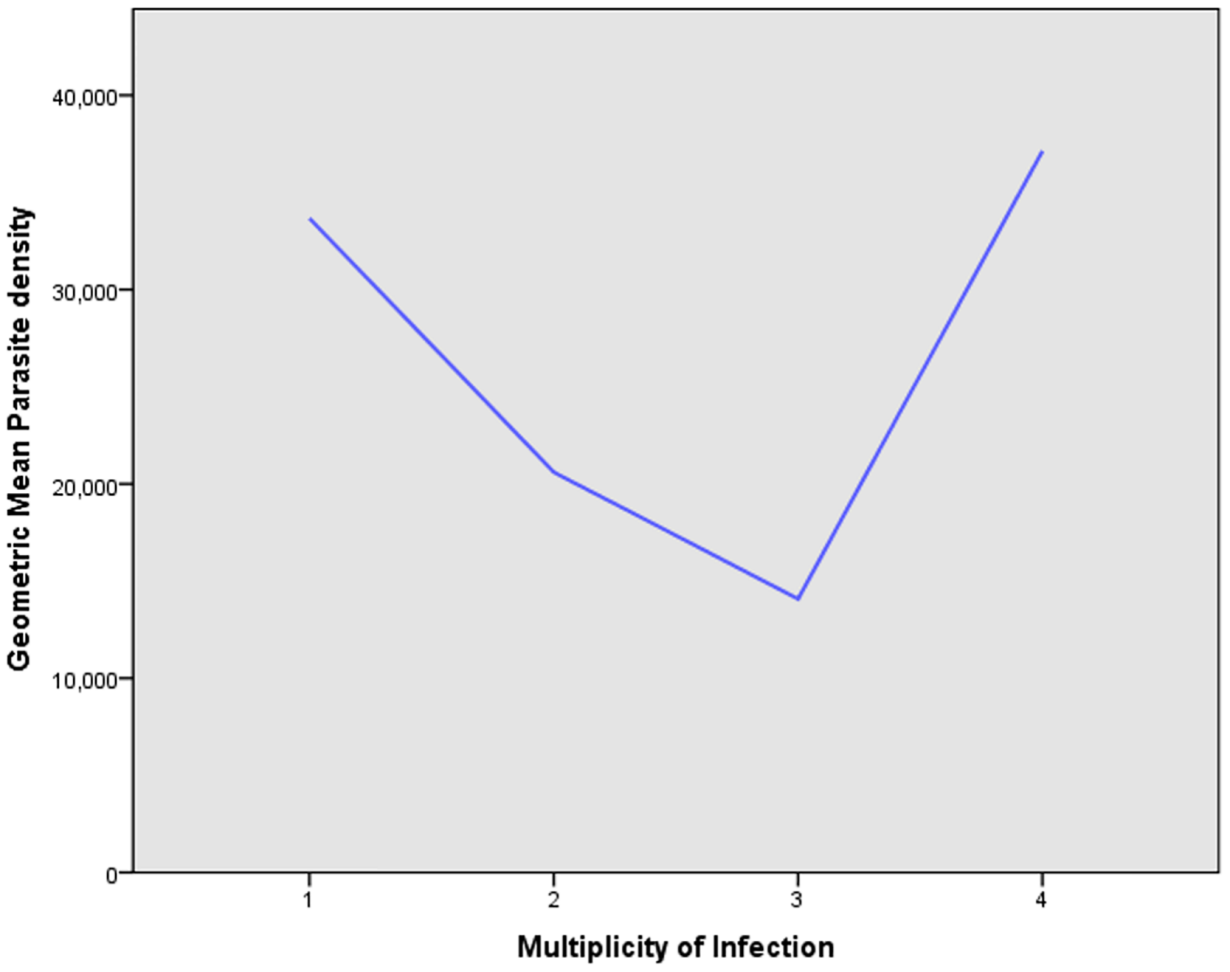
Relationship between geometric mean parasite density and multiplicity of *P*. *falciparum* infection (n = 92).

Alleles were determined by size and family type. A total of 22 different alleles were detected, of which 11 alleles belonged to FC27 type (300–700 bp) and 11 alleles belonged to 3D7/IC type (250–650 bp) (Figs 3 and 4). The proportion ofisolates havingonly3D7/IC and FC27alleleswas 12(13%)and 10(11%) respectively. Sixty-eight(74%)ofthe isolates carried bothallelicfamilies. Twoper cent (2%) ofthe isolateswerenot identified to anyofthe3D7/ICorFC27allelic familydespite being repeatedly amplified. Seventy-six percent (70/92) of the isolates contained multiple *msp2* alleles and the overall mean multiplicity of infection was 2.8 (CI 95%2.55–3.03). No statistical difference was observed between multiplicity of infection according to age or parasite density (P = 0.411) as shown in Figs 2 and 5. Expected heterozygosity was 0.66 for the *msp2* locus. Ninety isolates that successfully identified for the presence of MSP2 allelic families (FC27 and 3D7/IC) were classified according to the size of their amplified fragment (S1 and S2 Figs).

## Discussion

Compared to other countries, limited studies have been carried in Ethiopia exploring the genetic diversity of *P*. *falciparum*. To our knowledge this is the second such study, after our previous report from southwest Ethiopia [16]. This study determined the genetic polymorphism of *P*. *falciparum* in the Pawe area, using the most polymorphic region of the *msp2* gene. A high genetic diversity of the population of *P*.*falciparum* isolates from the study area was found. These findings may also be an important indicator to inform the effect of control interventions in the country.

The allele specific *P*.*falciparummsp2* genotyping showed that the malaria parasite population in the Pawe area is highly diverse. However, fragments with interval lengths of less than 20 base pairs could not clearly be distinguished as separate alleles. Among the isolates of msp2 diversity, a predominance of 3D7/IC allelic family was found in study area. This has also consistent with reports from Congo Brazzaville [20], sub-Saharan Africa [13] and Cameroon [22]. but differsfrom genotyping in Benin [23] and Sudan [24]. This suggests that the diversity of *P*. *falciparum* infections may differ according to geographical location, transmission intensityand samplepopulation.

The heterozygosity in the present study was relatively high (He = 0.66), suggesting a large genotype diversity within the *msp2* locus, which is higher than previously reported in Ethiopia and Malalysia [16,25]. In contrast, declining rates of diversityofalleles(heterozygosity)in *P*.*falciparum* are associated with decreasing transmissionrates [26,27]. The current study area has high genetic diversity in parasite populations with high local transmission and thus it requires increased attention with malaria control programs.

Furthermore, the present study demonstrated that three quarters of the isolates (76%) harbored multiple genotypes. This suggests a relatively high diversity of the *P*.*falciparum* parasite population in this localized area. This finding is similarto reports fromthe RepublicofCongo [20] and BurkinaFaso [28]. This finding is consistent with an association between malaria transmission levels and the frequency of multiple infections, with individuals living in high malaria transmission areas generally harboring multiple parasite strains.

Apparent multiplicity of infection (MOI) is often used as proxy for transmission and higher MOI reported in some areas may be related to thetransmission intensity [29]. The MOI reported in this study was high compared to those reported from a moderate transmission region in Kolla-Shele Southwest Ethiopia [16], but consistentwith findings from Cote d’Ivoire [30]. Thisisinagreement withprevious observations of an increased MOIwith increase ingendemicity [31]. As reported in Sudan [32], thehighrateofMOIinthepresent studysuggeststhat the scale-up of malaria control interventions may have failed to reduce the level of MOI, and this is contrast the malaria control efforts. Additionally, determination of the entomological inoculation rate is important toidentify the malaria transmissionlevels and the extent of MOI. In the present study, it is difficult to estimate the effect of mosquito inoculums on the multiplicity of infection with *P*. *falciparum*, due to unavailability of entomological data in the study area. The genetic diversityand MOI reported in the present study confirms a high level of local malaria transmission.

The results of this study show thatage has no associationon multiplicity of infection, similar to reports from others studies [33]. Onthecontrary, this finding contrasts with reports from Brazzaville, Republic of Congo [34]. This suggests that the effect of age on the multiplicity of infection is not directly related to the periodic acquisition of immunity. Previous studieshave showna correlationbetween the MOI andparasitedensity [20], but in our study the MOI did not increase with higherparasite densities, similarto reportsfromotherstudies [35]. The results of this study showed that isolates from younger children had higher parasite densities compared to adult patients. This is in agreement with previous reportsfrom Sudan [23]. These findings suggest that immune status may bea determining factor in the observed variations.

The present study results shows high allelic diversity in *P*. *falciparum* isolates in the study area, however, the *msp2* gene has immunologic effects under selective pressure [36]. Moreover, this study was performed in a single blood sample may however, not enough to reveal the whole diversity of the parasite population harbored by an individual, and the use of a single marker that may under estimate the magnitude of multiple infections [37, 38]. Further longitudinal studies, using neutral markers such as microsatellites or single nucleotide polymorphismsmay be betterfor evaluation of the parasite populations’ dynamics.

## Conclusion

This study of *P*.*falciparum* isolates from northwest Ethiopia, found isolates to have ahighgeneticpolymorphismand that most infections were from multipleclones. This result underlines the fact that assessment of parasite diversitycan play an important role in the evaluation of malaria control interventions. Therefore, furtherinvestigationindifferent transmission setting is required toassess the changes in the genetic parameters and to evaluatemalaria control interventions in the country.

## Supporting information

S1 Fig Electrophoresis separation of MSP2 genotyping with FC27 allelic types. (DOCX)

S2 Fig Electrophoresis separation of MSP2 genotyping with 3D7/IC allelic types. (DOCX)

S1 Table Data file document. (XLSX)

## Abbreviations

BP: base pairs
EPHI: Ethiopian public health institute
He: heterozygosity
IAEA: International atomic energy agency
MOI: multiplicity of infections
msp2: merozoite surface protein 2 gene
PCR: polymerase chain reaction
WHO: world health organization
