# Supplementary material for: Genetic polymorphism of Merozoite Surface Protein-2 (MSP-2) in Plasmodium falciparum isolates from Pawe District, North West Ethiopia
Source: PLoS One. 2017 May 19;12(5):e0177559. doi: 10.1371/journal.pone.0177559 (PMC5438166; doi:10.1371/journal.pone.0177559)
Supplement: S2 Fig — (DOCX) [file pone.0177559.s002.docx]

## S2 Fig. PCR genotyping of 3D7/IC with positive control


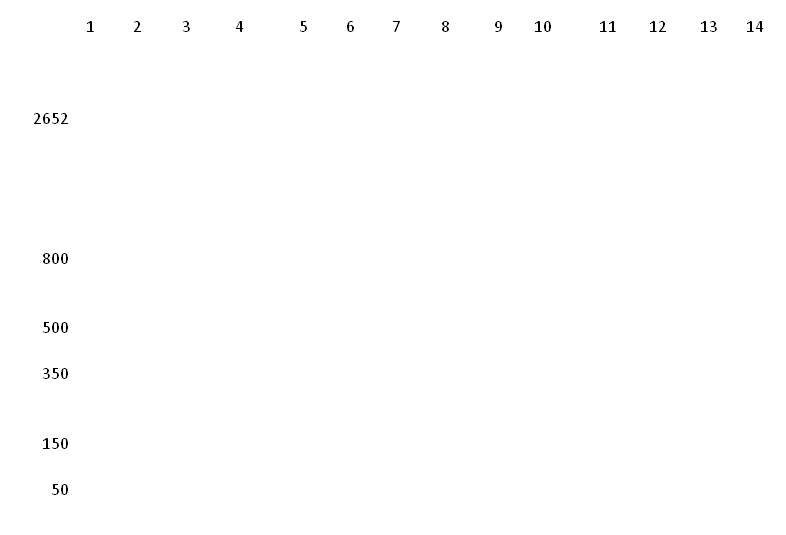

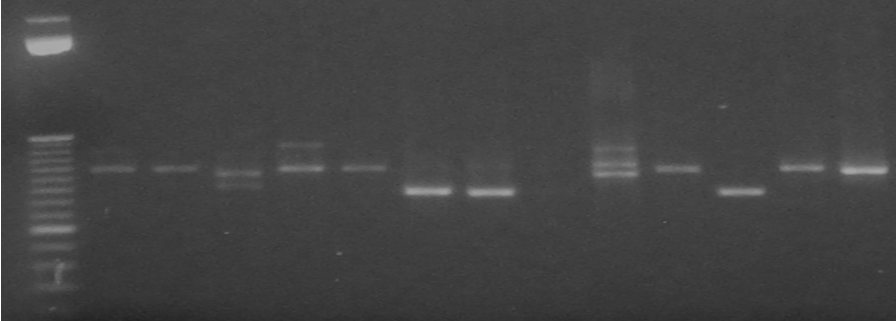


1 2 3 4 5 6 7 8 9 10 11 12 13 14

500

350

150

2652

**S2Fig. Electrophoresis separation of MSP2 genotyping with 3D7/IC allelic types**: Lane 1 50-base pair ladder in 2% agarose gels, lanes 2 and 3 (550bp), lane 4 (450-500 bp), lane 5 (550-700bp), lane 6 (550bp), lanes 7 and 8 (300bp), lane9 negative control, lane 10 (450-700bp), lane 11 and (450bp), lane 12 (450bp), lane 13 (550bp), lane 14 positive control.
